# Comparison of pain, cortisol levels, and psychological distress in women undergoing surgical termination of pregnancy under local anaesthesia versus intravenous sedation

**DOI:** 10.1186/1471-244X-7-24

**Published:** 2007-06-12

**Authors:** Sharain Suliman, Todd Ericksen, Peter Labuschgne, Renee de Wit, Dan J Stein, Soraya Seedat

**Affiliations:** 1 MRC Research Unit on Anxiety and Stress Disorders, Department of Psychiatry, University of Stellenbosch, Tygerberg, Cape Town, 7505, South Africa; 2 Marie Stopes Clinic, Cape Town, 8001, South Africa

## Abstract

**Background:**

The weight of evidence suggests that women who freely choose to terminate a pregnancy are unlikely to experience significant mental health risks, however some studies have documented psychological distress in the form of posttraumatic stress disorder and depression in the aftermath of termination. Choice of anaesthetic has been suggested as a determinant of outcome. This study compared the effects of local anaesthesia and intravenous sedation, administered for elective surgical termination, on outcomes of pain, cortisol, and psychological distress.

**Methods:**

155 women were recruited from a private abortion clinic and state hospital (mean age: 25.4 ± 6.1 years) and assessed on various symptom domains, using both clinician-administered interviews and self-report measures just prior to termination, immediately post-procedure, and at 1 month and 3 months post-procedure. Morning salivary cortisol assays were collected prior to anaesthesia and termination.

**Results:**

The group who received local anaesthetic demonstrated higher baseline cortisol levels (mean = 4.7 vs 0.2), more dissociative symptoms immediately post-termination (mean = 14.7 vs 7.3), and higher levels of pain before (mean = 4.9 vs 3.0) and during the procedure (mean = 8.0 vs 4.4). However, in the longer-term (1 and 3 months), there were no significant differences in pain, psychological outcomes (PTSD, depression, self-esteem, state anxiety), or disability between the groups. More than 65% of the variance in PTSD symptoms at 3 months could be explained by baseline PTSD symptom severity and disability, and post-termination dissociative symptoms. Of interest was the finding that pre-procedural cortisol levels were positively correlated with PTSD symptoms at both 1 and 3 months.

**Conclusion:**

High rates of PTSD characterise women who have undergone surgical abortions (almost one fifth of the sample meet criteria for PTSD), with women who receive local anaesthetic experiencing more severe acute reactions. The choice of anesthetic, however, does not appear to impact on longer-term psychiatric outcomes or functional status.

## Background

Despite the high rate of elective abortions, controversy still exists about the psychological risks associated with this procedure. On the one hand, while the weight of evidence suggests that when women freely choose to terminate a pregnancy they are unlikely to experience significant mental health risks [1-3], some studies have documented psychiatric disorders such as posttraumatic stress disorder (PTSD) in the aftermath of termination [4-6]. Several factors have been associated with post-termination responses, including type of abortion procedure and age. With regards to age some studies have found that younger age may be associated with a higher risk of negative responses post-abortion [7,8], while others that have compared psychological responses in adolescents under 18 years of age with those over 18 have shown no differences [2].

Notably, no significant differences in anxiety or depressive symptoms have been documented in women undergoing medical or surgical termination [9,10]. However, in women who undergo surgical termination, awareness during anaesthesia especially if it is associated with pain, [11] may be so traumatic so as to place some women at risk of developing PTSD [12]. Seeing the foetus may be traumatic [7], while awareness or consciousness during experiences of blood, pain and death (of the foetus) have also been associated with PTSD-type symptoms [10], namely re-experiencing (cycle of intrusion and denial), avoidance (numbing) and perception of the termination as traumatic [6].

Several studies, mostly retrospective, have described the development of PTSD in patients who experience awareness under anaesthesia for various surgical procedures [13-15]. However, findings have not been altogether consistent. For instance, Slade et al. [10] documented more postabortion distress (including nightmares, flashbacks, and unwanted thoughts related to the procedure) in women who received general anaesthesia (GA) (with presumably no or little intraoperative awareness) compared with women who received local anaesthesia (LA).

The trend to perform surgical abortions under LA rather than GA is growing, with many clinics now providing supplementary medication in the form of conscious/intravenous sedation (IS) (i.e., opioid analgesia) in an effort to improve pain control. However, the benefit of IS in women undergoing abortions is unclear. It has been noted that women who receive IS report less subjective distress and pain than those who receive LA [16,17]. On the other hand, it has been suggested that IS improves patient satisfaction but not pain [18,19]. For example, Rawling & Wiebe [20] and Wiebe [21] found no or questionably significant relief in women who received IS.

In view of the lack of prospective studies on introperative and postoperative effects of pain and awareness on long-term psychological, social and physical well-being, this study was conducted to examine the effects of elective surgical pregnancy termination on mental and physical health status when IS versus traditional LA techniques are used.

We aimed to determine if either LA or IS predicted posttraumatic stress disorder, depression, anxiety or physical ill health in women at 1-month and 3-month follow-up, and to examine other predictors (e.g. pain, self-esteem, age of woman, gestational age, cortisol), if any, of post-abortion PTSD at 3 months. We hypothesized that women who received IS would have significantly less pain, PTSD, depression and anxiety symptoms compared with women who received no sedation. Based on previous findings, we also expected baseline cortisol levels to be inversely correlated with PTSD at 3 months. PTSD is known to be characterized endocrinologically by an alteration of hypothalamic-pituitary-adrenocortical (HPA) axis function with lowered cortisol secretion [22]. There is evidence, too, for a longitudinal inverse correlation between PTSD symptoms and morning salivary cortisol levels [23], although this has not been well characterized.

## Methods

### Subjects

155 consecutive referrals (80 from a private abortion clinic in Cape Town and 75 from a state-funded secondary level general hospital with an obstetrics/gynaecology unit) were recruited. Referrals were women undergoing surgical termination of pregnancy under local anaesthesia or sedation. Both sites offered local anaesthesia and sedation and the choice of anaesthetic was determined by patient preference. To be eligible, women had to be 16 years of age and older, be at 6–20 weeks of gestation, have an unintended pregnancy that was not the result of rape, and be fluent in English. Women were invited to participate at the time they received counselling for their termination.

The mean age of participants was 25.4 years (range: 16 – 41 years; SD: 6.1 years), with 34% of women in the 16 to 21 year age group. Age differences were evident between the anaesthetic groups, with women in the IS group significantly older than women in the LA group (27.5 ± 6.6 years vs. 24.2 ± 5.4 years, t = 3.2, *p *= 0.002).

There were no significant group differences with respect to ethnicity, marital status, number of children, previous miscarriages, previous abortions, or previous assault history. Most women were of mixed ethnicity [Coloured] (48.3%) and were living with a partner (55.6%). 51.7% of participants had one or more living children. 59.0 % had had an abortion prior to this one and 12.6% had previously miscarried. Approximately 42% of women had experienced some type of assault in their lives, with the most common forms of assault being mugging (10.6%), rape (9.3%) and domestic violence (9.3%).

### Procedure

This study was conducted with the approval of the Committee for Human Subjects at the University of Stellenbosch, and was in compliance with the Helsinki Declaration. Written, informed consent was obtained from all subjects prior to enrolment. Participants were assured that their responses would be confidential and anonymous, and that refusal to participate would in no way jeopardise their management. Eligible women were interviewed at 4 time points. A pre-termination assessment (T1) was conducted approximately 1–3 hours prior to the procedure and prior to administration of anaesthetic. The termination procedure followed (this varied in duration from 5 to 35 minutes). Follow-up questionnaires were then completed in the recovery room just prior to discharge (T2), then at 1 month (T3) and at 3 months (T4) post-procedure. All assessments were done in person, except for the 3-month assessment (T4) which was telephonic. A Demographic Questionnaire was devised for the study and included information on age, ethnicity, religion, marital status, education, socio-economic level, prior medical/surgical/psychiatric history, prior live births, prior abortions. The research psychologist who conducted the assessments was blind to the anaesthetic status of subjects.

### Anaesthesia

The local anaesthetic used at both centres was Petercaine 1% injection (i.e. 10 mg per ml of lignocaine hydrochloride monohydrate). In addition, women at the state hospital received pre-medication comprising indomethacin (100 mg suppository) and diazepam (10 mg orally). The sedation was done using one of two combinations: Propofol 1% (20 ml) + Alfentanyl 1 mg (2 ml) + Ketamine 20 mg (2 ml) *or *Propofol 1% (20 ml) + Ramifentanyl, administered intravenously. The induction dose was 0.85 mg per kg (Propofol) and increased as necessary to achieve the desired effect. Duration of administration varied according to patient needs and duration of procedure. All patients (local anaesthetic and sedation) received Misoprostol (400 mg/800 mg/1200 mg) and those undergoing sedation also received Midazolam 7.5 mg ± 2 hours orally pre-op. Use of these methods was dependent on the locale of the procedure, approaches of the providers, wishes of, and affordability to the participant.

### Instruments

The following were administered at the pre-termination assessment (T1):

#### 1. Visual Analogue Scale [VAS] for pain

The VAS consists of a 10 cm line with the endpoints 0 ('no pain') and 10 ('worst pain').

Participants are asked to indicate their current pain intensity on the scale using a cross (X). The VAS is a widely used measure of pain intensity and has also been shown to have adequate sensitivity to change in pain associated with treatment across many populations and settings [24].

#### 2. Clinician-Administered PTSD Scale [CAPS-1]

The CAPS [25] has become the 'gold' standard for outcome studies in PTSD. It provides current and lifetime PTSD diagnostic information in addition to frequency (0 = never; 4 = daily or almost every day) and severity (0 = none; 4 = extreme) scores of individual PTSD symptoms. Participants are asked to respond to the questions with reference to the previous week and with a particular trauma in mind. participants are asked to respond to the questions with reference to the last week and a particular trauma in mind at T1, and with reference to the termination as the index trauma at T3 and T4.

The recommended score of ≥ 50 was used to identify women with PTSD.

#### 3. Davidson Trauma Scale [DTS]-self report

The DTS [26] is self-rated measure of PTSD comprising 17 items that correspond to each of the DSM-IV symptoms of PTSD. The respondent rates frequency (0 = never; 4 = daily or almost every day) and severity (0 = none; 4 = extreme) for each item on a 5-point scale. A cut-off score of 40 on the DTS is considered to be indicative of PTSD. As with the CAPS, participants are asked to respond to the questions with reference to the last week and a particular trauma in mind at T1, and with reference to the termination at T3 and T4.

#### 4. Spielberger State-Trait Anxiety Inventory [STAI] state scale

The STAI [27] is a self-rated scale that measures current anxiety symptoms on a scale of 1 to 4 (1 = almost never; 4 = almost always). A score of ≥ 40 is considered 'high anxiety'. The State version of the STAI was used.

#### 5. Beck Depression Inventory

The Beck Depression Inventory (BDI) [28] is a self-rated measure of depressive symptoms. Respondents choose from 4 possible answers ranging in intensity from 0 to 3. As a screening test for depression, recommended cut-off points are: 0–9 non-depressed, 10–15 mild depression; 16–23 moderate depression; and ≥ 24 severe depression.

#### 6. Rosenberg Self-Esteem Inventory

The Rosenberg Self-Esteem Inventory (RSI) is a well-validated and widely used measure of self-esteem [29]. Respondents indicate how they feel on scales of 1 (strongly agree) to 4 (strongly disagree). A higher score is indicative of a better sense of self-worth. The range of possible scores is 10 – 40, with mean scores below 20 indicating average responses on the negative side of self-esteem, and mean scores above 30 indicating average responses on the positive side of self-esteem [30].

#### 7. Schneier Disability Scale (SDS)

The SDS assesses current and lifetime impairment in 8 domains of functioning and each item is rated separately for current and most severe lifetime disability on a 5-point, descriptively anchored scale ranging from 0 (no impairment) to 4 (severe impairment). The item scores may be totaled to obtain 2 summary scores, one rating overall current disability and the other most severe lifetime disability. Alternatively item scores may be considered individually to provide descriptive information on the pattern of impairment across domains.

Immediately post-procedure (T2), the following measures were administered: *(i) Visual Analogue Scale (VAS) for Pain and (ii) Dissociative Experiences Scale-Taxon [DES-T]*. The DES-T, derived from the 28-item Dissociative Experiences Scale [DES] [31], is an 8-item subscale designed to discriminate pathological from non-pathological forms of dissociation [32,33]. The scale focuses on pathological changes in consciousness, such as dissociative amnesia, depersonalization and derealization. Subjects are asked to allocate a percentage of time that they experience each type of dissociative phenomenon (0% – 100%).

All assessments done pre-termination (except for the demographic and pain assessments) were repeated at the 1-month (T3) & 3-month (T4) follow-up visits.

Data from 151 participants were included in the final analysis (78 from the private clinic and 73 from the state hospital). Four patients were excluded owing to missing data at T1 (pre-termination). Of the 151 participants, 76 % (N = 115) completed assessments immediately post-procedure, 44 % (N = 67) returned for the 1-month assessment and 37 % (N = 56) for the 3-month assessment.

Thirty-seven percent of the sample received intravenous sedation (IS) while 63% received local anaesthetic (LA).

### Salivary cortisol

Morning (09 h 00) saliva specimens were collected at pre-termination (T1). Samples (approximately 5 ml volume) were collected and stored at -20 degrees celsius, until assay. Samples were analysed using a commercial radioimmunoassay.

### Ethical issues

Study clinicians did not participate in counselling, ethical decision-making, or termination procedures and adhered to stringent anonymity and confidentiality requirements. In part, owing to anonymity and confidentiality issues involved in following a termination sample over time, some of the original sample was lost to attrition.

### Statistical analyses

The two groups (LA] vs. IS) were compared on primary outcome measures (VAS for pain, CAPS for PTSD) in addition to other demographic and clinical variables, using chi-square tests (for categorical variables) and student's t-tests (for numerical variables). Possible associations between the groups were also analysed, controlling for other variables. Women were also stratified by age (16–21 and >22 years), and chi-square and student's t-tests were used to compare responses between the groups. For outcomes measured across time (e.g., PTSD, depression, self-esteem, physical health scores), and in comparing baseline differences of those who dropped out of the study with those who completed it as well as gestational age of women with and without PTSD at 3 months, mean differences between the groups were analysed using student's t-tests. Correlations were examined among the different outcomes (e.g. PTSD, depression, self-esteem) using Pearson correlation coefficients. The DTS, BDI, and Spielberger scales were categorized into high and low scores (determined by each scale's recommended cut-off) to determine thefrequency of cases above the cut-off for clinical significance. In order to determine whether or not there were any statistically significant differences between groups over time, we performed GLM Repeated Measures ANOVAs. The influence of demographic/clinical characteristics on PTSD outcome was examined by simultaneously entering variables that emerged as statistically significant from bivariate analyses into a multiple linear regression equation to predict PTSD scores at 3-months. The significant variables were then entered into a stepwise regression which produced 3 models. The semi-partial correlation coefficients and betas were computed in order to evaluate the unique contribution of each predictor variable. For all analyses, statistical significance was set at *p *<.05 and all tests 2-tailed.

## Results

### Differences between LA and IS groups

#### (i) Pain

Participants who received IS reported less pain, (i.e., significantly lower pain scores) than those who received LA both at baseline (IS: 3.0 ± 2.3; LA: 4.9 ± 3.1; *p *= 0.000) and during surgery (IS: 4.4 ± 3.0; LA: 8.0 ± 1.0; *p *= < 0.005).

#### (ii) PTSD

No significant differences in PTSD symptom status (CAPS and DTS total scores) were observed between the groups at either the 1 month or 3 month assessments. At baseline, 11.3% of the sample met criteria for a diagnosis of PTSD on the CAPS (IS: 7.3%; LA: 14.1%), 17.5% met criteria at 1 month (IS: 9.4%; LA: 28.0%), and 18.2% at 3 months (IS: 10.0%; LA: 28.0%). No significant association between group status (IS/LA) and the three month CAPS scores was found.

#### (iii) Other psychiatric outcomes

Women who received IS experienced significantly less dissociation immediately following the procedure than women who received LA (IS: 7.3 ± 11.3; LA: 14.7 ± 17.6; *p *= 0.01). With respect to longer-term outcome, there were no significant differences in depression, self-esteem, state anxiety, and disability scores between IS and LA groups at 1 and 3 months.

With respect to depressive symptoms, at pre-termination 21.9% of the sample had scores suggestive of clinical depression (IS: 21.8%; LA: 23.1%), while at both 1 and 3 months, 20% of women (IS: 13.8%; LA: 25.7%) had scores suggestive of clinical depression. 63.9% scored 'high' on state anxiety (STAI) at pre-termination (IS: 66.0%; LA: 62.6%), while at both 1 and 3 months, 56.3% of women (IS: 51.7%; LA: 60.0%) had 'high' STAI scores.

Secondary analyses were conducted to compare women with and without a PTSD diagnosis on pain and psychiatric symptoms at 1 and 3 months follow-up. Women with a PTSD diagnosis on the CAPS endorsed significantly more pain after the procedure at both 1 month (With PTSD: 7.0 ± 3.3; Without PTSD: 3.9 ± 2.5; *p *= 0.049), and 3 months (With PTSD: 8.0 ± 2.4; Without PTSD: 3.7 ± 2.4; *p *= 0.012). They also had higher CAPS, DTS, anxiety, and disability scores at 1 month. Notably, those who met PTSD criteria at 3 months after termination had significantly lower depression (With PTSD: 6.8 ± 6.7; Without PTSD: 27.8 ± 8.2; *p *= 0.003) and self-esteem (With PTSD: 23.0 ± 1.9; Without PTSD: 31.9 ± 6.1; *p *= 0.000) scores at one month than those who did not meet criteria. GLM Repeated Measures ANOVAs for baseline, 1 and 3 month CAPS scores, baseline, 1 and 3 month DTS scores, and baseline, 1 and 3 month BDI scores comparing anaesthetic status were performed, while controlling for age, baseline cortisol, and prior trauma exposure. Results indicated that there was no significant differences between groups (IS/LA) over time. However there were significant differences between pre-termination and 1 month DTS scores (F = 4.52, df = 4,18, *p *= 0.03) and 1 and 3 month BDI scores, F = 16.40, df = 2,18, *p *= 0.00). There were no significant baseline differences between participants who dropped out of the study prior to completion and those who completed it. However, women with PTSD 3 months after termination were further along in their pregnancy than those without PTSD (gestational age: With PTSD: 13.2 ± 3.3; Without PTSD: 9.7 ± 4.2; *p *= 0.023).

#### (iv) Age

At baseline, younger women (16 – 21 years) endorsed more depressive symptoms (Younger: 16.2 ± 9.1; Older: 12.7 ± 9.0; *p *= 0.027) and had lower self-esteem scores than older women (> 21 years) (Younger: 27.2 ± 6.0; Older: 30.3 ± 5.6; *p *= 0.003). They also, reported significantly more pain post-termination (Younger: 5.3 ± 3.1; Older: 3.7 ± 2.8; *p *= 0.011) and had higher anxiety scores at 1 month (Younger: 48.2 ± 8.9; Older: 41.9 ± 11.9; *p *= 0.026) and 3 months (Younger: 48.7 ± 9.0; Older: 41.9 ± 11.9; *p *= 0.021).

#### (v) Salivary cortisol

Women in the IS group had significantly lower baseline cortisol levels than those in the LA group (IS: 0.2 ± 0.2; LA: 4.7 ± 17.6; *p *= < 0.001). Cortisol levels were positively correlated with baseline pain (r = 0.5; *p *= 0.000) and state anxiety (r = 0.3; *p *= 0.006) and negatively correlated with baseline self-esteem (r = -0.4; *p *= 0.009). At 1 and 3 months, baseline cortisol correlated positively with total CAPS scores (i.e. higher baseline cortisol levels were associated with more PTSD symptomatology) (r = 0.3; *p *= 0.043). Additionally, women with "high" anxiety scores at pre-termination and 1 month after the procedure had higher baseline cortisol levels than those with "low" anxiety scores (High anxiety: 0.5 ± 0.6; Low anxiety: 0.3 ± 0.2; *p *= 0.024).

In summary, women who received IS reported significantly less pain before and during the procedure, and had less severe dissociation and lower baseline cortisol than women who received LA.

#### Predictors of PTSD at 3 months

To determine which variable/s, if any, independently predicted CAPS PTSD scores at 3 months, variables that emerged as statistically significant in bivariate analyses, namely PTSD (CAPS) scores at baseline, disability at baseline (Schneier) and post-termination dissociation (DES-T) were entered as predictor variables into a forward stepwise linear regression analysis. This procedure produced three models. The first model retained baseline CAPS scores and accounted for 44.7% (adjusted R^2 ^= 0.43) of the variance in 3-month CAPS scores. The second model retained CAPS and disability scores at baseline and accounted for 57.1% (adjusted R^2 ^= 0.54) of the variance in PTSD symptoms. The third model retained CAPS and disability scores at baseline and post-procedural dissociation. This model accounted for 66.1% (adjusted R^2 ^= 0.62) of the variance in PTSD symptoms. The semi-partial correlation coefficients and betas were also computed in order to determine the unique contribution of each predictor variable.

## Discussion

Our results do not demonstrate any notable long-term differences in outcome between the two groups (sedation/LA). Thus, PTSD, depression, state anxiety, and disability were not associated with the type of anaesthesia received. Although not statistically significant, women who underwent local anaesthesia were more likely to meet PTSD criteria.

The CAPS was used to establish a diagnosis of PTSD based on traumatic event exposure prior to termination and, at subsequent assessments, a diagnosis of PTSD secondary to the termination procedure. The point prevalence of PTSD at baseline was 11.3%, which is comparable with US community norms [10] but lower than the rate obtained in another local community study [34]. The prevalence of PTSD after termination was 17.5% and 18.2% at one and three months respectively. This is higher than the rate found by Rue et al. [5] in American and Russian women (14.3% and 0.9% respectively), but lower than that found in other studies of pregnancy loss, for example, by Engelhardt et al. [35]. Of note, PTSD symptom status at 3 months was predicted by baseline (pre-termination) severity of PTSD symptoms and the level of pain experienced, but was not predicted by the presence of other psychopathology (depression, state anxiety, self-esteem, and functional disability. Women with PTSD were more likely to be further along in their pregnancies. This has been noted in studies by Grimes and Cates [36] and Davies et al. [37], and has been explained on the basis that women in an earlier stage of pregnancy have had a shorter relationship with the foetus, are less likely to have experienced foetal movements and, as such, may be at lower risk. In addition, high levels of pre-existing PTSD and functional disability, as well as post-termination dissociation appear to contribution to PTSD in the longer term in this population. Thus it would follow that screening women pre-termination for PTSD and disability and post-termination for high levels of dissociation is important in order to help identify women at risk of PTSD and to provide follow-up care. Both dissociation [38] and pre-existing PTSD [39,40] have been identified as risk factors for PTSD in other trauma populations.

Baseline rates of depression (21.9% had scores indicative of clinical depression) and general anxiety (63.9% had high STAI scores) were also high, with prevalence rates similar to those found in other studies. For example, in their review, Bradshaw and Slade [41] observed that prior to termination, levels of 'caseness' on measures of anxiety, depression and overall psychological distress ranged from 15 to 69%. Anxiety scores in the present study are also comparable with those documented in pre-operative samples [42]. However, rates of depression, and state anxiety (as measured on the DTS, BDI and Spielberger, respectively) at post-termination did not differ significantly from pre-termination rates. Rates of depression were similar to population based rates in the US [8] but much lower than found previously in a local sample (Carey et al., 2003) [34]. Similar to the findings reported here, several other studies have found lower levels of depression [7,43,8]) and lower state anxiety scores [44] post-abortion compared with pre-abortion.

The finding of lower baseline pain levels in women who received IS is consistent with observations by Wells [16] and Rawlings and Weibe [17,20]. At post-termination, the LA group reported higher levels of dissociation than the IS group. It could be argued that higher levels of pain in the LA group might have contributed to a greater propensity to dissociate. For example Duckworth et al. [45] observed that rates of dissociation were generally higher in persons with pain, even among "non-traumatised" patients.

Higher cortisol levels in the local anaesthesia group were consistent with the greater experience of pain. Other studies, for example Zimmer et al. [46], have also shown significant correlations between pain and cortisol levels. Additionally, in this sample higher pre-procedural cortisol levels were positively correlated with PTSD symptoms at 1 month and 3 months. The correlation between cortisol and pain and cortisol and anxiety in this study may further be representative of the association between anxiety and pain as suggested by Belanger, Melzack & Lauzon [47] and Taenzer [42]. However, whether higher baseline cortisol is an etiologic marker of PTSD symptom severity or is merely an epiphenomenon of some other process remains unclear and warrants further investigation.

Pain intensity has been significantly correlated with subjective distress [16], including stress and anxiety [48] and it has been suggested that the experience of pain during termination cannot be separated from the anxiety and trepidation that are experienced before the procedure [19]. In fact, the level of pre-abortion anxiety has been positively associated with pain experienced during abortion [47] and is consistent with the role of anxiety in pain perception and response [42]. Consistent with findings from previous investigations [16], pain and PTSD symptoms tended to co-occur in the sample.

There are several limitations to this study. First, there was a high rate of attrition over the course of the study leaving a small final sample (37% of the original sample). It might be that participants who were lost to follow-up were lost because of their higher levels of postabortion distress (i.e. PTSD and other psychopathology), resulting in lowered rates of psychiatric disorder, although our results indicated no baseline differences between those who dropped out and those who completed the study. Second, salivary cortisol samples, although collected in the morning (within a two hour window) were not all collected at the same time, and there was no control for other variables known to affect cortisol (time of last meal, caffeine consumption, smoking status). Fluctuations in cortisol may have confounded these results and thus should be interpreted very tentatively. Third, this was not a randomised study as the participants were referred to the study only after selecting the type of anaesthetic they would receive. Fourth, sedation was not equally available as a treatment option across both sites and this may have, to some extent, biased these results. For example, the use of ketamine may change the way in which patients remember the procedure. Fifth, the 3-month follow-up was conducted telephonically, contrary to the other 3 assessments, and responses may be partially accounted for by the method of assessment. However, responses at the 1-month and 3-month assessments remained very similar. Sixth, the majority of participants were of mixed ethnicity (coloured) and while this is the majority ethnic group in the province, these results may not be generalizable to other ethnic groups.

## Conclusion

High rates of PTSD characterise women who have undergone voluntary pregnancy termination. The type of anaesthetic for the termination procedure does not seem to impact on long-term outcomes, however women who receive IS appear to fare better on pain and post-termination dissociative phenomena.

Presently the weight of evidence suggests that abortion does not cause lasting negative consequences [1,49]. Nevertheless, longitudinal assessment of multiple variables (e.g., pain, type of termination, anaesthetic procedure, gestational age, cortisol, trauma-related symptoms and other psychophysiological markers) that may potentially impact on subsequent PTSD and major depression is deserving of further study.

## Abbreviations

Beck Depression Inventory: BDI; Clinician Administered PTSD Scale: CAPS; Davidson Trauma Scale: DTS; Dissociative Experiences Scale- Taxon: DES-T; General Anaesthesia: GA; Hypothalmic-pituitary-adrenocortical: HPA; Intravenous Sedation: IS; Local Anaesthesis: LA; Posttraumatic Stress Disorder: PTSD; Rosenberg Self-Esteem Inventory: RSI; Scheneier Disability Scale: SDS; Spielberger State-Trait Anxiety Inventory: STAI; Visual Analogue Scale: VAS

## Competing interests

The author(s) declare that they have no competing interests.

## Authors' contributions

S Suliman drafted the manuscript and participated in the design, co-ordination and data collection of the study. TE helped to draft the protocol and participated in its design and data collection. PL conceived of the study. RDW participated in data collection. DJS helped to draft the manuscript. S Seedat drafted the protocol, designed the study, performed the statistical analysis and helped to draft the manuscript. All authors have read and approved the final manuscript.

**Table 1:** Comparison of Clinical Outcomes in Women receiving Intravenous Sedation (IS) versus Local Anaesthetic (LA)

		**Pre-termination (N = 151)**		**Post-termination (N = 115)**		**1 – month (N = 67)**		**3 – month (N = 56)**	
		Mean	*p*	Mean	*p*	Mean	*p*	Mean	*p*

**PTSD (CAPS)**	IS	9.2 ± 22.7	0.355	N/A		16.1 ± 26.2	0.074	17.2 ± 26.8	0.107
	LA	12.9 ± 24.1				29.2 ± 27.4		29.2 ± 27.4	
**PTSD (DTS)**	IS	42.2 ± 32.5	0.067	N/A		30.3 ± 30.4	0.712	30.3 ± 30.4	0.701
	LA	32.4 ± 26.2				33.2 ± 31.1		33.3 ± 31.6	
**Depression (BDI)**	IS	14.5 ± 9.8	0.602	N/A		10.2 ± 9.5	0.29	10.2 ± 9.5	0.279
	LA	13.6 ± 8.8				13.0 ± 12.1		13.1 ± 12.2	
**Self-esteem (Rosenberg)**	IS	29.8 ± 6.2	0.331	N/A		30.9 ± 6.3	0.175	30.9 ± 6.3	0.152
	LA	28.8 ± 5.7				28.7 ± 6.7		28.5 ± 6.8	
**Anxiety (Spielberger)**	IS	44.4 ± 12.0	0.645	N/A		41.1 ± 10	0.149	41.4 ± 10.7	0.142
	LA	43.4 ± 10.6				45.5 ± 11.7		45.6 ± 11.9	
**Disability (Schneier)**	IS	4.8 ± 4.6	0.138	N/A		2.8 ± 4.0	0.327	2.8 ± 4.0	0.401
	LA	3.6 ± 3.2				3.9 ± 4.3		3.8 ± 4.3	
**Cortisol**	IS	0.2 ± 0.2	0.001	N/A		N/A		N/A	
	LA	0.5 ± 0.5							
**Dissociation (DES-T)**	IS	N/A		7.3 ± 11.3	0.01	8.1 ± 13.0	0.142	8.1 ± 13.0	0.213
	LA			14.7 ± 17.6		14.4 ± 20.4		13.3 ± 19.7	

• CAPS: Clinician-Administered PTSD Scale

• DTS: Davidson Trauma Scale

• BDI: Beck Depression Inventory

• DES-T: Dissociative Experiences Scale-Taxon

**Table 2:** Variables Demonstrating a Significant Association with PTSD Status at 3 months

**Variable**	**PTSD**	**No PTSD**
	
	**3 month mean and SD**	
Baseline PTSD (CAPS) score*	52.6 ± 30.7	7.5 ± 17.8
Post-abortion pain score*	8.0 ± 2.4	3.9 ± 2.8
1 month PTSD (CAPS) score**	81.6 ± 14.3	11.5 ± 16.9
1 month PTSD (DTS) score**	93.0 ± 17.7	20.9 ± 19.7
1 month Anxiety (STAI) score**	58.4 ± 3.3	39.5 ± 8.6
1 month Depression (BDI) score**	6.8 ± 6.7	27.8 ± 8.2
1 month Self-esteem (RSI) score**	23.0 ± 1.9	31.9 ± 6.1
1 month Disability (SDS)**	8.8 ± 2.2	2.89 ± 4.3

*: *p *< 0.05

**: *p *< 0.01

**Table 3:** Partial Correlations and Standard Beta- values

	**Variables**	**Standard Beta**	**Partial Correlation**	***P***
**Model 1**	Baseline PTSD (CAPS) Score	0.668	0.668	0.000
**Model 2**	Baseline PTSD (CAPS) Score	0.473	0.413	0.003
	Baseline Disability score	0.404	0.353	0.011
**Model 3**	Baseline CAPS Score	0.432	0.357	0.004
	Baseline Disability Score	0.389	0.339	0.007
	Post-termination Dissociation	0.303	0.299	0.017

## Pre-publication history

The pre-publication history for this paper can be accessed here:


